# Observations on Digitalis, with Cases of Consumption and Hydrothorax

**Published:** 1806-10

**Authors:** W. Hamilton

**Affiliations:** Halesworth


### Observations on Digitalis, with Cases of Consumption and Hydrothorax. By Dr. Hamilton.

( Continued from our last, pp. 229—234. )

IN hydrothorax "there is great difficulty of breathing, and the patient cannot rest in bed, unless the head and the upper part of the trunk be more or less eleva ed from the horizontal posture. The sleep is often suddenly interrupted by alarms and disagreeable dreams; the urine is in very small quantity, and there is commonly anasarca of the legs. The pulse is generally irregular; but this is not always the case. There is a paleness in the countenance, with a purple hue of the lips and of the cheeks, if the latter should happen to have any tinge remaining. This change of colour is produced by the veinal blood not passing readily through the lungs, which are prevented from expanding themselves sufficiently for this purpose by the accumulation of the water." Drowsiness and coma also frequently arise from the same cause; and the impossibility of procuring sleep often occasions delirium.

To this enumeration of symptoms from Dr. Baillie, it may be added, that this disease seldom appears till late in life, that it most frequently attacks males, and such as have been addicted to spirituous potation; it is also distinguished by a look of peculiar anxiety, and by a ghastly countenance. The difficulty of breathing is at times much increased, accompanied with convulsive action of the muscles of respiration, similar in appearance to periodic asthma. The patient cannot exist without the free access of fresh air; cough and expectoration of frothy mucus are also frequent symptoms of hydrothorax. Persons afflicted with the gout are peculiarly liable to this disorder.

It may be doubted whether there are any particular symptoms, by which, the presence of water within the different cavities of the chest, can be distinctly ascertained.

Irregularity of the pulse is supposed to denote the pericardium to be the seat of the disease; but this generally happens in all cases of hydrothorax. The author of Zoonomia supposes, that anasarca pulmonum may be distinguished, by the greater facility experienced by patients labouring under this disorder, in assuming the recumbent posture, and by the power of digitalis in effecting its cure; while, where the water is confined within the cavities of the chest, he seems doubtful of its efficacy. In one of the following cases however, (Mr. Hayles) the power of continuing in the horizontal posture was but little diminished, yet there was scarcely any œdematous swelling of the ancles, and in them all, the digitalis had a most salutary effect.

It is observed by Dr. Cullen, that hydrothorax occurs more frequently than has been imagined. And if anasarca pulmonum is included in the general term, there can be, I apprehend, no doubt of the justice of the remark.* In the humoral asthma, and chronic cough affecting old persons, we very frequently observe a swelling of the feet and legs, and a diminished secretion of urine. To its power in removing this disposition, by its action on the kidneys, especially if its use has been aided by small doses of calomel, rather than to any expectorating quality it possesses, I presume squills to be indebted. In all such cases an effusion of water into the cells of the lungs may perhaps with great reason be suspected, and to them the observation of Baglivi, "in morbis pectoris semper ducendum esse ad vias urinæ," is peculiarly applicable. Here digitalis will seldom fail to procure relief, and its use ought to be persevered in, according to the urgency of the symptoms. And I believe with Dr. Maclean, that "it will be found highly

* Bree on Disordered Respiration, p. 69 et seq.

highly beneficial in asthmatic affections, coughs of every kind, dyspnœa, and other chronic affections of the chest and lungs." *

A collection of water in any of the cavities of the chest constitutes a disease supposed to be attended with imminent danger; and indeed the importance to life, of the functions carried on by the parts thus affected, must render their being impeded, particularly hazardous. Doctor Cullen observes, "this disease seldom admits of a cure or even of alleviation." To this character of hydrothorax it may perhaps have been owing, that less is usually attempted for its relief, than in most other instances of severe disease, and like some other predictions therefore, it may thus have contributed to its own confirmation. The following case is copied from a late author, in support of this opinion. It will shew how this disease is sometimes managed even by the most eminent physicians.

A gentleman, under forty, after suffering severely from gout, "found his breath become short at night, and his strength much impaired; and at the end of a fortnight the breathing became so difficult at going to bed, as to require his being propped up in a sitting posture every night. He now applied for medical aid; when his pulse being found to be very low, and he exceedingly weak, and no shortness of breathing taking place in the day time, a blister was applied between the shoulders, and various antispasmodics and expectorants were tried for a few days, without success. He was then bled, and lost about five ounces of blood, from which he experienced very great relief to his breathing; but, at the same time, suffered a considerable farther prostration of strength. In about four or five days, the shortness of breathing returning to as great a degree as before, it was proposed to take away three or four ounces of blood, *by cupping*, from the chest. But although the shortness of breathing was exceedingly distressing, yet he begged to avoid the loss of more blood, confident that, although it might afford him temporary relief, the exhaustion of his strength would be irreparable. All that was prescribed by the eminent physician who had first seen him, as well as by two others, proved ineffectual, and within six weeks he sunk, overcome by the accumulation of water in the chest." †

* Medical and Physical Journal, vol. ii. p. 117.
† Parkinson on Gout, p. 63.

But it would be very unfair, although much weight must be attached to the conduct of three eminent physicians in consultation, to conclude from the treatment in this unfortunate case, that so little is attempted towards effecting the cure of hydrothorax in general. Notwithstanding the commonly received opinion of the fatality of this disease, much, no doubt, is often endeavoured to be done, and the common remedies of dropsy, generally exhibited. I apprehend however that these will commonly fail, and I fear I am justified in concluding, that the fox-glove, which appears by the following cases to have so decided a beneficial influence upon this species of dropsy, is not often applied to for its cure, or is inefficaciously administered. I am not acquainted with any work in which it is recommended, except in Dr. Maclean's paper already quoted.* Dr. Ferriar does not lead us to expect much from it in this disease. And Dr. Darwin, who speaks so highly of its powers in anasarca pulmonum, is doubtful of its efficacy in hydrothorax, and submits as a question, "Is it ever cured by making the patient sick by tincture of digitalis? † Should I however be mistaken in this opinion, which forms my principal inducement for calling the attention of your readers to this subject, I still hope the following cases may not prove entirely deficient in interest.

In exhibiting this remedy in hydrothorax, I have followed the proportion of Dr. Withering's infusion, of ʒj of the dried leaves, carefully prepared, to ʒviij of water, which has appeared to me the most efficacious mode of exhibition in dropsy; of this infusion, from ʒv to ʒviij with some aromatic tincture, have been usually given every sixth or eighth hour, in proportion to the urgency of the case, and the strength of the patient; and the dose has been increased, till the desired effect was produced. I have very generally, at the same time, prescribed one or more grains of calomel, which I suppose to quicken the action of all diuretic medicines, daily. This effect however has commonly taken place, long before the calomel could be supposed instrumental in producing it. No attention has been paid to Dr. Withering's directions, with regard to the choice of patients, as I have given this medicine in every instance of hydrothorax in which I have been for some time past consulted, and the following cases form

* Medical and Physical Journal, vol. iv. p. 127.

† Zoonomia, vol. iii. p. 168.

form the whole of my experience of its use, in this disorder. Two cases only have I suppressed, as affording no information. In one the patient could not be prevailed upon to persist in the use of the medicine, although it was evidently beneficial; and in the other, organic disease was combined with the effusion of water.

Mr. Warner of Framlingham, aged under fifty, accustomed to considerable exercise in the superintendance of a large farm, and not addicted to the use of spirits, was attacked in the spring of 1801 with the symptoms of hydrothorax, and becoming gradually worse, notwithstanding a variety of medical advice, I was desired to see him on the 15th of May. I found him propped up with pillows upon a sofa, and unable to remain a moment in the recumbent posture; his countenance exhibited much of the peculiar anxiety generally found in this disorder, the pulse was rapid and intermitting, in so great a degree, that eight or ten pulsations were frequently lost; the urine was high coloured, and scanty, and the ankles swelled. A purge was given, and he was directed to take ℥vj of infusion of digitalis in an aromatic draught every six hours, and a grain of calomel, night and morning. No nausea or sickness followed the use of the medicine, which produced a very considerable flow of urine, and all his symptoms were thereby relieved. In a few days he was able, with little inconvenience, to walk about his farm, and in less than three weeks he rode to my house, a distance of sixteen miles, on horseback, before breakfast; some strengthening medicines were now given him, and Mr. Harsant of Wickham Market, a very ingenious surgeon, who attended him with me, was requested to observe him frequently, and to have recourse to the digitalis again, should any symptom of the former affection appear.

This attention was not unnecessary, for in no long time the urine again became scanty, the legs swelled, and difficulty of breathing, upon exertion, again occurred. He was now relieved by the digitalis as before, and he was requested to apply immediately to Mr. Harsant should his symptoms at any time return. He did so, two or three times before his death, which occurred February 6, 1804; but although his faith in the medicine was unbounded, he became tired of applying for it. He had for some time previous to his death, suffered from his usual symptoms, though not so severely as formerly, and to a friend who urged his application to Mr. H. he replied, he would soon, if he did not get better; on the morning of his death he was shaving himself, and expired instantaneously.

Mr. Welton, of Yoxford, aged sixty-five, a largely employed builder and auctioneer; whose situation in life and convivial disposition, equally led him into company, and who has drank no common quantity of wine and spirits, has for many years been troubled with swellings of the ankles and legs, frequently also the thighs; and the lungs, several times, have partaken of the general affection. Mr. W. has also for many years suffered from gout. In 1800 I visited him, under symptoms denoting a considerable degree of hydrothorax; but although, from the rapidity of his recovery, I have little doubt that he took the digitalis, yet as I have no notes of the case, and the death of the surgeon who attended him with me, having soon after taken place, I cannot obtain any certain account of the mode of treatment adopted, and I relate this part of the case merely to shew the period, from which this disposition has existed.

In 1803 I saw him again; his legs were now much swelled, the urine scanty, cough and dyspnœa urgent, and he was entirely unable to bear a recumbent posture; the pulse in this case did not intermit, it was about 100, small and hard. The digitalis was given, as in the former case, and increased till some degree of nausea took place, and a fortnight's perseverance in its use relieved all the symptoms; they have since returned twice, and have been by the same means removed, and this patient continues to enjoy as good health as he has done for many years past. It is worthy of remark, that Mr. W. formerly drank wine only, from an apprehension of the injurious tendency of spirits. Of late years he has almost entirely confined himself to spirits, in which he has indulged very freely. Since this change in his liquor, he has been much less afflicted with gout, as perhaps, from the late discoveries of Doctor Pearson, Doctor Wollaston, &c. respecting the formation of the uric acid, might be expected.

On October 15, 1803, I was desired to visit Mr. Hayle of Marlesford, with Mr. Harsant, his son-in-law. This patient was between sixty and seventy, and had many years freely indulged himself in spirituous potation. In the spring of 1802, he had the influenza in London, by which he was greatly debilitated, and he attributed his illness to the effects of that complaint. I found him labouring under excessive difficulty of breathing, with severe fits of coughing and expectoration of frothy mucus. His pulse was was rapid and intermitting, in a very great degree; there was great paleness and anxiety of the countenance; the urine was scanty, but the ankles were very little swelled; and although he could scarcely bear the slightest degree of exertion, he was able to continue in the horizontal posture, without much inconvenience: A proof that the most characteristic symptoms of this disease are sometimes absent. An ounce of the infusion of digitalis, prepared as above, was ordered to be given him every sixth hour, with a grain of calomel night and morning; but it being sent in a half-pint mixture, a practice, when active medicines are employed, highly improper; as he found considerable benefit after the third dose, he determined to increase the quantity. He accordingly took two doses of, I suppose, nearly two ounces each, allowing however the interval of six hours to elapse between them, which produced all the deleterious effects of an over dose of this plant. On the 7th of October I was summoned to him in great haste, as he was believed to be dying. I found him labouring under excessive sickness, and that he had brought off his stomach a large quantity of watery fluid, though he had drank nothing; the pulse was reduced to 60, and was much more regular, and the difficulty of breathing, the cough, and other symptoms of hydrothorax, were entirely gone.

The sickness was relieved by opium, acids, and aromatics; and a more guarded use of the digitalis, after a proper interval, was persisted in. The symptoms of hydrothorax did not return, and in a very short time he resumed his usual occupations. Within a fortnight he was able to ride over his farm, and in a month he performed a journey of nearly forty miles in a day, on horseback. In this case, notwithstanding the relief obtained, no considerable increase of the urinary secretion took place till after the sickness had subsided. With his accustomed occupations, however, his usual habits also returned, and his propensity to indulgence was freely gratified; his former symptoms again appeared, and more than once he was obliged to apply to Mr. Harsant, for some of his medicine, which never failed to relieve him.

In April 1805, he was deprived of the use of one side and entirely confined to his bed by a paralytic attack, and I saw him soon after in consequence. He had now no difficulty of breathing, œdematous swellings of the legs, or scarcity of urine, and his pulse, which had never been entirely free from intermission, was much as usual. It was suspected that water might be effused upon the brain, and with this view, mercury, digitalis, and some other remedies were tried, but without the appearance of benefit, and he died suddenly in the beginning of August following.

In the winter of 1805, I was called up in the night to Mr. Favre of Yoxford, who was believed by his attendants to be dying. I found him under a severe paroxysm of difficult breathing, and his pulse labouring, quick, and intermitting; he had not been able to lie down for several nights, and he was delirious; his legs and thighs were enormously swelled, and his urine in very small quantity. Mr. Favre was seventy-three years of age, and had been many years accustomed to the free indulgence of his taste for spirits, insomuch that he scarcely ever for a considerable time was known to go sober to bed. I prescribed for him the digitalis in the usual manner, and his relief from it was very striking, though not quite equal to what I had before experienced. He was obliged to continue its use for a considerable time, and although it created a great increase in the flow of urine, and the difficulty of breathing became much less urgent, he was not entirely relieved till his mouth was affected by mercury. He then speedily recovered from all his remaining symptoms of disease, and his strength gradually was restored. He spent the summer very comfortably notwithstanding his great age, and cultivated his garden as usual, but no remonstrances could prevent his indulgence in the habit of drinking. At the approach of winter his symptoms returned, and he died of universal dropsy, in spite of every means likely to afford him relief. As there was in this case considerable yellowness of the skin, with clay coloured fæces, I suppose the liver to be diseased.

Mr. Tilney is a very respectable school-master at Harleston in Norfolk; he is sixty-nine years of age, has lived very temperately, and has used little exercise except at times, when he has greatly exerted himself to perform long journies on foot, to which mode of travelling he was particularly attached.

In last spring Mr. Tilney was attacked with difficulty of breathing, cough and expectoration of frothy mucus; his ancles swelled, and he was unable to use any considerable exertion. He took squills, mercury, digitalis in small doses, &c. under the direction of a physician, without benefit, and his symptoms having alarmingly increased, I was desired on the 15th of May last to visit him. In making this visit I was accompanied by Dr. Fothergill, to whom your Journal is so much indebted for the able Reports of the the Diseases of the Westminster and Western Dispensaries, then on a visit with me, and we met Mr. Strowger, an intelligent surgeon of the place, who carefully attended this patient through the whole of his illness. We found Mr. Tilney propped up in bed with pillows, and labouring under the most distressing degree of difficulty of breathing. He was gasping for breath, and the violently convulsive action of the muscles of respiration, seemed frequently to threaten immediate suffocation. His pulse was very quick, and intermitted so much, that it could hardly be reckoned, and he had got no sleep for many hours, and was delirious; the secretion of urine was almost suppressed, and his legs were œdematous.

In this case, which appeared almost decided, it required all my confidence in the salutary powers of digitalis, to enable me to excite any hopes of its probable success in the friends of my patient, or indeed, to feel them myself. It was however given, as the only means that could be at all likely to procure relief. He began with half an ounce of the infusion, which was afterwards increased to ℥vj; the bowels were opened with calomel, of which also he was directed to take a grain night and morning. On the 18th a considerable amendment was observed, which had gradually taken place; the infusion produced some nausea, the urine was increased, and the breathing considerably relieved; the pulse had less intermission, and was reduced to 90; his delirium was almost gone off, and he was able to get some comfortable sleep in his chair. On the 22d, the pulse was 54, and regular; swelling of the ancles and delirium gone, secretion of urine plentiful. He continued the use of the fox-glove another week, when every symptom of his former complaint had disappeared. He now was recommended strengthening remedies, of which he stood much in need, and about three weeks afterwards when I saw him, he assured me that he felt no remains of indisposition, except that he found he could not walk as formerly, without fatigue. He is now in perfect health.

The following case was communicated to me by Mr. S. Revans, an intelligent young surgeon of this place:

"Elizabeth Davy, of Rumburgh near Halesworth, aged sixty-five, was in April last attacked with dyspnœa, which daily became more oppressive, notwithstanding the use of a variety of medicines. In the beginning of June I was desired to see her; her dyspnœa was now so distressing, that she had not been able to go to bed for a fortnight, and and for the last week she had scarcely slept at all, even in an erect posture. Her pulse was labouring, and intermitted on the slightest exertion; she likewise complained of a sense of suffocation, and frequent and copious sweats broke out upon her forehead and face; her legs were much swelled, and the secretion of urine was greatly diminished.

"From a review of the symptoms of this case, I had no doubt of its being hydrothorax, and being acquainted with the event of Mr. Tilney's case, I determined to give the digitalis a fair trial. I made an infusion of a drachm of the dried leaves in half a pint of water, of which I directed her to take a table spoonful every six hours, and which after a few doses was increased to a table spoonful and half at a time. After her continuing the medicine for forty-eight hours it produced considerable giddiness and nausea, and she then voided a large quantity of urine, which she continued to do, particularly during the night, for a week, when she was completely relieved of the dyspnœa and all her distressing symptoms; and could walk and attend to her business with more ease than she had been able to do for the last two years.

"About a month back, she had a slight return of the difficulty of breathing, which was removed by the infusion in two or three days, and I have seen her within a week, entirely free of complaint."

I shall not detain you with any comments upon the above cases.

I am, &c.

*Haleswoorth, July* 14, 1806. W. HAMILTON.

