# Errata

**Published:** 1806-11

**Authors:** 


### *ERRATA.*

P. 356, line 16 from bottom, for ʒviii. read ℥viii.

P. 360, line 16 from bottom, for suppose, read supposed.

